# Large recurrent sialoliths in a residual Wharton duct after sialoadenectomy: Two case reports and literature review

**DOI:** 10.1097/MD.0000000000041231

**Published:** 2025-01-10

**Authors:** Li-Wen Su, Huan Sun, Hong-Yu Zhang, Yang Wu

**Affiliations:** a Department of Oral and Maxillofacial Surgery; b Department of General Dentistry, School and Hospital of Stomatology, Wuhan University, Wuhan, China.

**Keywords:** case report, recurrence, salivary calculi, salivary ducts, submandibular gland

## Abstract

**Rationale:**

When gland-preserving treatments are unsuccessful, sialoadenectomy is typically conducted for patients afflicted with submandibular gland diseases. The definitive treatment modality for these individuals is the removal of both the gland and the associated ducts. During surgery, the gland and the majority of the ducts can be excised utilizing the lateral transcervical approach, with residual ducts unlikely to develop pathology. After sialoadenectomy, the recurrence of salivary gland stones is extremely rare. Although there are some relevant speculations, to the best of our knowledge, there are no comprehensive reports of larger recurrent stone-related cases available.

**Patient concerns:**

We present 2 instances of recurrent sialoliths in the residual Wharton duct following sialoadenectomy. In our cases, it was not until several years later that both patients presented with symptoms. The patients, a 51-year-old male and a 28-year-old female, presented with swelling and purulent discharge in the right floor of the mouth.

**Diagnoses:**

Computed tomography scans revealed irregular high-density masses in the floor of the mouth, indicative of sialolithiasis.

**Interventions:**

The intraoral incision exposed the recurrent sialoliths, which were successfully removed along with the residual duct.

**Outcomes:**

There were no complications in both cases.

**Lessons:**

This report aims to clarify potential mechanisms behind recurrent sialoliths in residual Wharton ducts after submandibular gland excision, warranting further investigation to improve patient management. New stones may form again in the residual duct even if the glands were removed. With the risk of recurrent sialoliths after resection of the gland, multiway preventive management can optimize outcomes.

## 1. Introduction

Sialolithiasis is a condition characterized by stone formation in the ductal system of salivary glands, with the submandibular gland (SMG) being the most commonly affected.^[1–7]^ In the past, operative removal of the glands was the recommended therapeutic approach for up to 40% of all cases following unsuccessful conservative treatment.^[8–13]^ However, due to advancements in technology and equipment, the rate of gland removal has significantly decreased to below 5%.^[13]^ Sialoadenectomy should only be performed when gland-preserving treatments fail or when repeated gland infections lead to chronic sclerosing SMG inflammation, SMG atrophy, or even loss of SMG function.^[14]^ Emission computed tomography (CT) can accurately evaluate the salivary gland’s uptake and excretion functions.^[15]^ Although SMG removal can be easily executed, complete duct removal is often challenging due to its proximity to the lingual nerve and sublingual gland (SLG) ducts.^[16]^ Concurrently, severe tissue adhesion around the stones may arise, resulting in residual Wharton duct presence after sialoadenectomy. It is generally believed that removing the glands and the stone-containing ducts is sufficient. However, we encountered 2 clinical exceptions. The purpose of this article is to provide more complete clinical evidence for exploring the optimal surgical approach and to offer a method to prevent stone recurrence after SMG excision by examining these 2 cases in conjunction with a comprehensive analysis of recurrent cases over the past 60 years.

## 2. Case presentation

### 2.1. Chief complaints

A 51-year-old male presented with swelling and purulent discharge in the right floor of the mouth. The second patient, a 28-year-old female, experienced swelling and pain in the right floor of the mouth, accompanied by purulent secretion and occasional bleeding.

### 2.2. History of present illness

The first patient sought treatment at our hospital for swelling and purulent discharge in the right floor of his mouth. The second patient reported swelling and pain in the right floor of her mouth, along with purulent secretion and occasional bleeding.

### 2.3. History of past illness

Upon reviewing the medical records, it was revealed that the first patient had undergone right SMG resection in our hospital 10 years ago due to right SMG duct stones, with a successful recovery after surgery. However, he was lost to follow-up until he presented more recently with recurrent obstructive symptoms. The second patient had undergone resection of the right SMG in an external hospital 7 years ago, after which she experienced tongue numbness. She was also lost to follow-up until presenting with recurrent obstructive symptoms.

### 2.4. Personal and family history

Both patients denied any history of previous disease.

### 2.5. Physical examination

Patient 1 presented with swelling in the right floor of the mouth, and palpation revealed hard objects. Patient 2 displayed no obvious abnormality in the right floor of the mouth; however, palpation revealed thickened duct-like tissue and reported tenderness.

### 2.6. Imaging examinations

**Figure 1.**
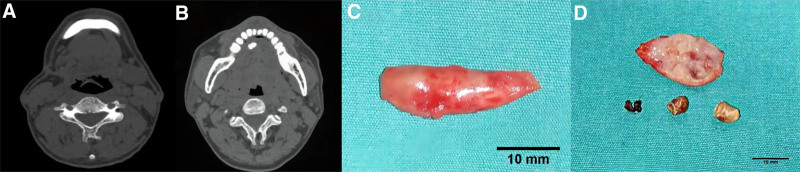
(A) Computed tomography (CT) shows the absence of a right submandibular gland in the patient 1. (B) CT shows 2 irregular high-density masses in the front part of the main submandibular duct in the right mouth floor area of patient 1. (C and D) Stones and suture were taken out during the operation of patient 1.

**Figure 2.**
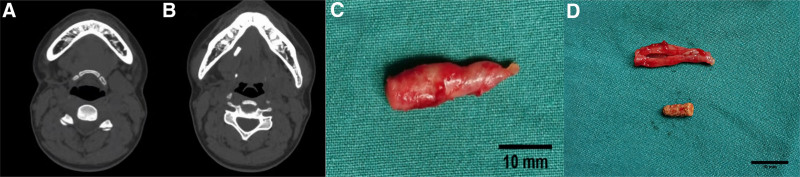
(A) Computed tomography (CT) shows the absence of a right submandibular gland in the patient 2. (B) CT shows the irregular high-density mass in the front part of the main submandibular duct in the right mouth floor area of patient 2. (C and D) Stones and suture were taken out during the operation of patient 2.

A CT scan for patient 1 revealed stones in the right SMG duct. Multislice helieal CT and three-dimensional reconstruction confirmed the absence of the right SMG, likely due to previous surgical removal. In addition, 2 irregular high-density masses with maximum diameters of ≈1.1 cm and 0.6 cm were visible in the right floor of the mouth anterior to the main SMG duct (Fig. 1A and 1B). For patient 2, multislice helieal CT and three-dimensional reconstruction revealed the right SMG had been removed, and the soft tissue in the right submandibular region was slightly swollen. Imaging also demonstrated a strip of high-density calcification, ≈1.3 × 0.5 × 0.6 cm in size, in the middle of the right SMG duct, with a nonuniform texture (Fig. 2A and 2B).

### 2.7. Final diagnosis

Based on patient history, clinical examination, and radiographic imaging results, the final diagnosis for both patients was recurrent sialoliths in a residual Wharton duct.

### 2.8. Treatment

**Figure 3.**
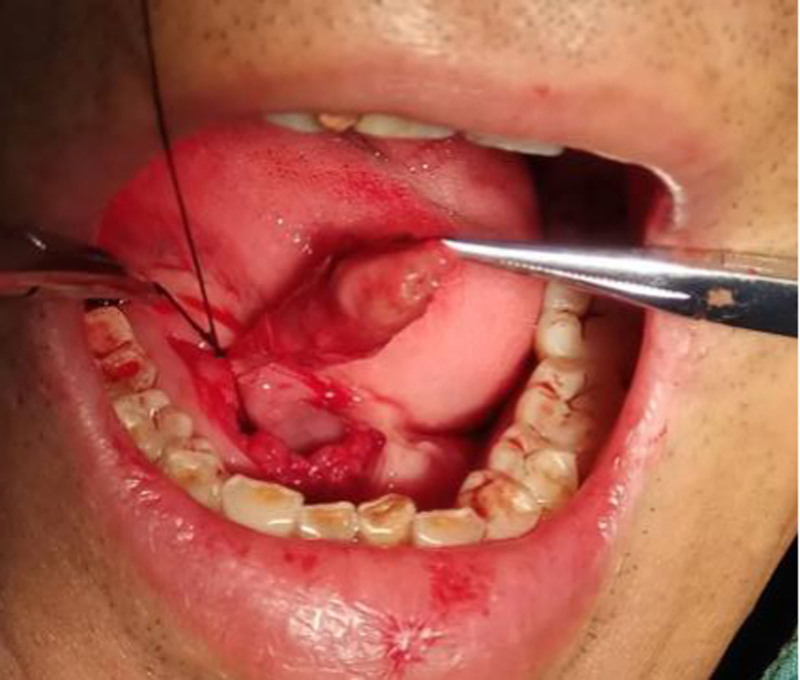
Intraoral image of the patient 1 during reoperation.

Following the diagnosis of recurrent sialoliths in a residual Wharton duct, both patients underwent surgical removal of the residual ducts and stones in the right submandibular area. The procedure was performed with informed patient consent and included routine disinfection and draping. Under local anesthesia, an incision was made in the right oral floor mucosa, the thickened SMG duct was located, and it was excised from the distal end of the previous operation’s suture. The entire duct was removed in 1 piece. After confirming no stone residue, the stump was ligated, and the wound was thoroughly irrigated. Hemostasis was achieved, and the wound was sutured and compressed to prevent further bleeding. In Figure 1, the stones and suture are visible upon cutting the removed duct from patient 1 (Fig. 1C and 1D). In Figure 2, the stone is visible upon cutting the removed duct from patient 2 (Fig. 2C and 2D). Intraoperative conditions were recorded (Fig. 3).

### 2.9. Outcome and follow-up

After surgery, both patients received anti-inflammatory treatment, and sutures were removed 1 week later. Both patients demonstrated successful recovery without any obvious abnormalities.

## 3. Discussion

Sialolithiasis is a prevalent condition, with postmortem studies indicating a 0.115% incidence of sialoliths in the general population, while the clinical (symptomatic) prevalence is ≈0.45%.^[3]^ A substantial 80% to 90% of sialolithiasis cases occur in SMG, with the distal duct and hilum being the most frequent sites of SMG stones.^[7]^ The exact etiology remains unknown; however, it is associated with salivary stasis, ductal inflammation, ductal injury, mucus plugs, and eventual calcification.^[17,18]^ Salivary gland calculi are more prevalent in adults than children, typically presenting between the ages of 50 and 80 years, with a higher prevalence in males.^[18]^ In 2022, a treatment algorithm was published incorporating all relevant therapeutic tools for sialolithiasis.^[19]^ Prognostic factors, including stone size, location, and mobility, have been acknowledged for their impact on treatment outcomes and have been considered in the new treatment algorithms. However, stone shape has not been accounted for.^[19,20]^ However, considering the possibility of stone recurrence, it is necessary to establish more standardized and rigorous treatment guidelines (preoperative diagnosis, intraoperative procedures, and postoperative follow-up) for clinical use. In 2009, a report summarized the results of ≈4691 cases treated in 5 different salivary gland centers using minimally invasive treatment measures over a 14-year period, with only 2.9% of patients requiring gland excision.^[21]^ It should be noted that initial stone removal approaches were less effective than current techniques, so present-day results are likely superior to those reported in the study. With current equipment and technology, gland-preserving treatment is feasible and can effectively address most cases of salivary gland stones. Nevertheless, in situations where gland-preserving treatment proves unsuccessful or results in the loss of salivary gland function, the removal of the gland and ducts remains a challenging option to circumvent. We generally assume that gland removal will permanently resolve the issue. Nevertheless, based on clinical observations, we have begun to question this perspective. In our cases, gland removal was necessitated due to the loss of gland function caused by SMG duct stones. Recurrent sialoliths following sialoadenectomy constitute a rare occurrence. Intraoperative omission is possible and frequently accompanied by symptomatic presentation or spontaneous stone discharge within a brief postoperative timeframe. However, in our cases, it was not until several years later that both patients presented with symptoms. Large stones, which are difficult to overlook during surgery, were found in the residual duct. We hypothesize that even after gland removal, new salivary stones may form in the residual duct. As early as 1968, Seward^[22]^ mentioned the recurrence of stones in their report, suggesting that postoperative recurrence may be due to residual glands or missed stones. They believed that saliva secreted by the SLG is less likely to contribute to stone recurrence. Subsequently, there have been occasional reports of similar cases, each with different speculations about the causes of recurrence. However, there is still very limited evidence regarding the reasons for recurrence. Large stones were discovered in the residual duct in their solutions. Furthermore, all reported cases have been rather vague, and there have been no systematic reports to date.

### 3.1. Search strategy and criteria

The English-language articles published up to September 1, 2023, were searched in the PubMed, Embase, and Cochrane databases. The comprehensive electronic search strategies included terms for SMG sialoliths (“submandibular” OR “salivary” OR “gland” OR “sialolithiasis” OR “sialolith” OR “megalith” OR “stone” OR “calculi” OR “calculus”) AND terms for operative technique (“sialoadenectomy” OR “excision” OR “removal” OR “resection”) AND terms for recurrent sialoliths in the residual duct (“recurrent” OR “residual” OR “remnant”). After removing duplicates, all studies were screened based on the title and abstract. In addition, the reference lists of all preselected articles were screened for further relevant papers.

We conducted a review of relevant literature spanning the past 60 years. Our literature search identified 71 articles based on the aforementioned electronic search strategies, with 60 retrieved from PubMed, 0 from Embase, and 11 from Cochrane. Of the 71 articles retrieved, 61 with irrelevant topics were excluded, leaving 10 articles^[22–31]^ that met the eligibility criteria. Relevant reports on recurrent sialoliths in a residual Wharton duct after sialoadenectomy are presented in Table 1.

**Table 1:** Review of recurrent sialoliths in a residual Wharton duct after sialoadenectomy, including our study.

The main author	Number of reported cases	The time of recurrence	The size of recurrent stones
Seward^[22]^	–	–	–
Coumel^[23]^	–	–	–
Milton^[24]^	3/134 (2.2%)	–	–
Patton^[25]^	3	Thirteen years after surgery, 9 years after surgery, and 11 years after surgery	Large
Berini-Aytes^[26]^	15/206 (7.3%)	–	–
Hald^27]^	8/44 (18.2%)	–	–
Ellies^[28]^	2/73 (2.7%)	A month after surgery and a year after surgery	–
Kim^[29]^	1	–	1.5 × 2 cm
Markiewicz^[30]^	1	Twelve years after surgery	11 × 4 mm, 4 mm
Ying^[31]^	1	Four years after surgery	–
This study	2	Ten years after surgery and 3 years after surgery	1.1 cm, 0.6 cm; 1.3 × 0.5 × 0.6 cm

### 3.2. Research advance in recurrent sialoliths in a residual Wharton duct

According to Seward,^[22]^ the recurrence of stones is postulated to result from residual glands persisting in saliva secretion or incomplete removal of stones from remaining ducts. It has been suggested that saliva secreted by SLG is less likely to contribute to the recurrence of stones. In 1979, Coumel^[23]^ emphasized the significance of systematically and comprehensively excising Wharton duct in the presence of calculi. This marked the first proposal advocating for such a procedure. Subsequently, in 1986, Milton^[24]^ hypothesized that rediscovered stones are residual remnants from the initial surgery. Contrarily, Patton^[25]^ proposed the possibility of stone reformation in the submandibular duct even after complete excision of the SMG. The 3 patients reported by Patton did not exhibit symptoms until 9 to 15 years post-SMG removal. The dimensions of the stones discovered during their diagnoses were too large to have been disregarded during the initial surgery, and in each case, the SMG had been entirely excised and the duct ligated. Patton proposed that these stones may have reformed due to saliva reaching the submandibular duct from the adjacent SLG, as communication between the sublingual glandular complex and the submandibular duct exists. However, this does not necessitate routine removal of the duct simultaneously, as it should be preserved to facilitate drainage of the major SLG and any posterior SLGs draining into the duct. Obstructive symptoms only manifest when calculi become large enough to impede residual submandibular duct drainage, compromising SLG function. Notably, in all 3 cases, SLG eventually necessitated removal and exhibited histological evidence of chronic sialadenitis. This supports the recommendation for meticulous “milking” of the submandibular duct posteriorly during surgery to expel any undetected calculi. Patton further suggested that if submandibular duct removal is required, consideration should be given to the associated SLG removal. The findings of Berini-Aytes and Gay-Escoda^[26]^ appear to bolster this perspective. They raised a controversial question, which is how much of Wharton duct should be removed. The development of a residual cyst in the mouth floor postsialoadenectomy can be attributed to either mucoceles (direct, partial injury of SLG) or mucous retention cysts (obliteration of Rivinus ducts by a 2-anterior ligature of Wharton duct). They believe that the reason why they did not observe any sublingual ranula is that the length of Wharton duct that they excised was usually short, which always left the sublingual drainage intact. Similarly, Kim et al^[29]^ also proposed that stones may have reformed due to saliva reaching the submandibular duct from the adjacent SLG. During the removal of residual ducts and stones, inflammatory adhesion between the ducts and SLG was identified, necessitating SLG removal alongside residual ducts. In 2015, Ying et al^[31]^ underscored the importance of retaining a portion of Wharton duct to avert SLG inflammation, recommending duct dilatation and plasty, as well as bottom-up duct massage for stone removal. Hald and Andreassen^[27]^ advised palpation of Wharton duct after gland extirpation, with imaging examinations suggested if complaints diverge from clinical findings. In 2007, Markiewicz et al^[30]^ posited that failure to remove Wharton duct during SMG resection may lead to persistent infection and calcification growth. There are reports emphasizing the importance of preserving a longer duct for SLG drainage. However, striking a balance between the 2 is crucial due to the risk of stone recurrence. Through a comprehensive literature review, various hypotheses regarding stone recurrence postsialoadenectomy were synthesized, culminating in the proposal of a multifaceted preventive management approach.

### 3.3. Risk factors

The precise mechanism of calculi formation remains elusive; however, it is likely that microscopic stones accumulate during normal salivary activity, creating atrophic foci that serve as proliferation sites for microbes ascending the primary salivary duct.^[32,33]^ According to a previous report, degenerative substances may be emitted by saliva through certain phenomena, with calcification around these substances potentially occurring, contributing to calculi formation.^[34]^ The incorporation of degenerative materials into the ductal system plays a crucial role in calculi development, with potential causative factors including inflammation, bacterial organisms, abnormal saliva stagnation, and foreign bodies.^[35]^ When the duct is obstructed due to various reasons, such as duct orifice stenosis, ductal inflammation, foreign bodies in the duct, or trauma to the mouth floor, saliva outflow becomes impaired, leading to a decrease in saliva flow rate. The expanded duct state causes calcium-rich material to stagnate and deposit around desquamated epithelial cells, foreign bodies, bacterial decomposition products, microorganisms, and/or mucus plugs, growing at a rate of 1 to 1.5 mm/year, ranging from 0.1 to 30 mm.^[32,36–38]^ In patients with recurrent stones post-SMG resection, saliva secreted by the SLG enters the residual submandibular duct (excluding a few cases where the SLG duct does not open into the SMG duct), providing a foundation for stone formation. In addition, prior stone presence suggests potential risk factors such as increased saliva viscosity and a decreased presence of substances in saliva capable of inhibiting calcium and phosphorus accumulation compared with healthy individuals.^[39]^ These risk factors are not mitigated by SMG resection. Research indicates that the saliva electrolyte composition of patients with sialolithiasis is significantly altered concerning crystallization mechanisms. Elevated calcium ion levels, acting as a crystallization substance, and reduced magnesium and citrate ions, serving as crystallization inhibitors, may contribute to the etiopathology of calculi formation.^[40]^ Furthermore, microliths have been identified in both normal and inflamed glands, and it has been postulated that they may constitute lith precursors.^[41–43]^ Microlithiasis can occur anywhere in the duct, and atrophic foci resulting from microliths would lack the flushing and bactericidal activity of saliva, thereby acting as a nidus for ascending bacterial establishment and proliferation. These bacteria would incite inflammation through invasion or waste product diffusion into surrounding tissues.^[44]^ Consequently, the longer the residual duct, the higher the likelihood of microlithiasis presence, and stones can develop on this basis. In addition, persistent inflammation may induce duct inner wall hyperplasia, causing ductal stenosis. The fibrous material generated by inflammation may obstruct microlithiasis discharge and even facilitate adhesion between microlithiasis and the duct inner wall, substantially elevating the risk of calculus recurrence. All of the aforementioned factors represent possible risk factors for patients with recurrent stones.

### 3.4. Multiway preventive management

**Figure 4.**
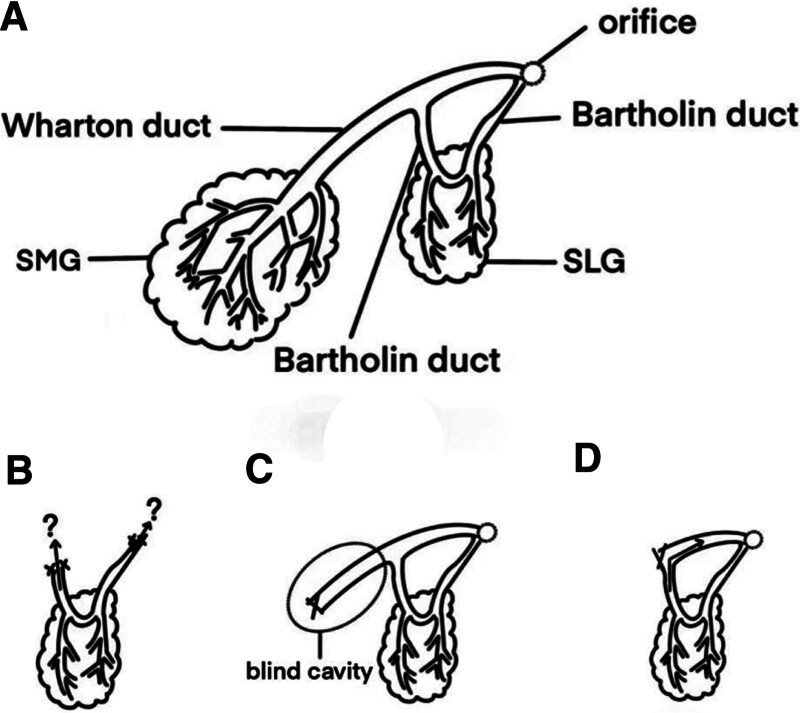
(A) The anatomical illustration of the ductal system of the submandibular gland, (B) complete removal of Wharton duct, (C) a blind cavity is formed between the ligated side of the residual Wharton duct and the Bartholin duct that merges into, and (D) cut and ligate the Wharton duct behind the confluence of the Bartholin ducts.

The treatment for sialolithiasis has undergone significant advancements due to the introduction of new devices, instruments, materials, and techniques. Techniques involving combined approaches have been refined and modified, with extracorporeal shock-wave lithotripsy, transoral duct surgery, and diagnostic and interventional sialendoscopy comprising substantial components of the contemporary treatment regimen. These methods boast high success rates and are less invasive than traditional surgical techniques, facilitating faster recovery times and decreased morbidity for patients. In recent years, intraductal shock-wave lithotripsy has gained increasing importance in sialolithiasis treatment, prompting an expansion in the scope of less invasive, gland-preserving treatment options. Although effective, intraductal shock-wave lithotripsy may not consistently offer faster treatment compared with alternative methods. Nevertheless, intraductal shock-wave lithotripsy demonstrates promising outcomes in terms of high success rates, minimal morbidity, and shorter patient hospital stays. Further investigation is required to ascertain the full potential of this technique in sialolithiasis management.^[19]^ Transoral robotic surgery has emerged as a safe and effective modality for managing hilar and intraparenchymal SMG sialoliths, exhibiting high procedural success rates regarding sialolith removal and SMG preservation. Transoral robotic surgery, a minimally invasive surgical technique employing a robotic system to access and remove sialoliths, offers numerous benefits, including reduced pain, minimized scarring, and expedited recovery times compared with traditional surgical approaches. However, additional research is needed to determine long-term outcomes and potential complications.^[3]^ Salivary gland removal should be contemplated for sialolithiasis cases resistant to other treatments, where there are persistent symptoms, and in accordance with the patient’s wishes. This option must be carefully balanced against the potential benefits of gland preservation, such as maintaining saliva production and circumventing surgical complications. Gland removal decisions should be made on a case-by-case basis, accounting for symptom severity and duration, sialolith size and location, and the patient’s overall health and preferences.^[18]^ Even when salivary gland removal is necessary, employing novel devices, techniques, and their combinations can effectively address missed stones during surgery. However, postoperative stone growth in the ducts remains a concern. Two key factors warrant consideration in reducing stone recurrence rates: the saliva secreted by the SLG and the residual Wharton duct. Two primary perspectives have emerged on managing the SMG and SLG ductal systems, with anatomical illustrations of the ductal system available to guide clinicians (Fig. 4A). One management approach entails complete duct removal; however, this method presents certain limitations. First, complete Wharton duct excision necessitates additional intraoral incisions, potentially increasing complication risks and prolonging the recovery period. Second, this approach may damage the SLG’s excretory duct (Bartholin duct) that opens into the SMG duct (Wharton duct), potentially affecting SLG drainage and precipitating further complications. Consequently, this method must be carefully evaluated and tailored to each patient’s unique circumstances (Fig. 4B). Theoretically, preserving SLG drainage post-Wharton duct removal requires careful attachment of the major Bartholin ducts to the floor of the mouth’s mucous membrane. However, this may prove challenging in practice. An alternative perspective posits that removing the gland and the stone-containing duct may suffice, as sialolith recurrence is rare and can be readily managed through intraoral incision if necessary. Nonetheless, it is crucial to consider the potential formation of a blind cavity between the ligated side of the residual Wharton duct and the converging Bartholin duct (Fig. 4C). Saliva is predisposed to retention in this area, considerably increasing the risk of stone recurrence. Consequently, we do not recommend either method for the aforementioned reasons. The crux lies in striking a balance between the 2 extremes. Our approach entails deliberately excising as much of the elongated Wharton duct as feasible by cutting and ligating it posterior to the confluence of the Bartholin ducts. This strategy aids in mitigating the risk of stone recurrence (Fig. 4D). For patients with an independent SLG duct opening in the sublingual caruncle, cutting and ligating the opening end of the Wharton duct suffice. This process results in the complete removal of the duct, with the remaining duct minimized to reduce stone recurrence likelihood. In addition, this method does not impede normal SLG discharge. It is essential to possess a robust understanding of stone recurrence potential postsialoadenectomy and to contemplate more advantageous surgical techniques capable of yielding superior long-term therapeutic effects. Of course, achieving this delicate balance of duct excision during the actual surgical procedure may be challenging. However, in order to reduce postoperative recurrence, it is worthwhile for surgeons to continually explore this approach while also anticipating the emergence of related surgical instruments and patent inventions. In addition to this, preoperative examinations and timely postoperative follow-ups should not be overlooked, as they can effectively reduce the occurrence of missed stones.

## 4. Conclusion

Our case emphasizes the challenges encountered in managing the Wharton duct during sialoadenectomy and highlights the possibility of new stone formation within the residual duct, even after gland removal. This underlines the importance of continuous management during the perioperative period. Although sialolith recurrence in a residual Wharton duct following sialoadenectomy is rare, it remains a consideration that must be acknowledged. The mechanisms underlying recurrent sialoliths are intricate, and no definitive conclusions have been drawn. However, based on a literature review and our clinical experience, cutting and ligating the Wharton duct posterior to the confluence of the Bartholin ducts not only significantly reduces stone recurrence but also ensures efficient SLG drainage.

## Acknowledgments

The authors wish to thank the participating patients.

## Author contributions

**Conceptualization:** Li-Wen Su

**Investigation:** Li-Wen Su

**Methodology:** Li-Wen Su

**Project administration:** Li-Wen Su

**Resources:** Li-Wen Su, Yang Wu

**Software:** Li-Wen Su

**Supervision:** Li-Wen Su, Yang Wu

**Validation:** Li-Wen Su, Yang Wu

**Visualization:** Li-Wen Su, Yang Wu

**Writing – original draft:** Li-Wen Su, Huan Sun, Hong-Yu Zhang, Yang Wu

**Writing – review & editing:** Li-Wen Su, Huan Sun, Hong-Yu Zhang, Yang Wu

## References

[1] Capaccio P Torretta S Ottaviani F. Modern management of obstructive salivary diseases. Acta Otorhinolaryngol Ital. 2007;27:161. 17957846 PMC2640028

[2] McGurk M Escudier MP Brown JE. Modern management of salivary calculi. Br J Surg. 2005;92:107–12. 15573365 10.1002/bjs.4789

[3] Rogalska M Antkowiak L Kasperczuk A Scierski W Misiolek M. Transoral robotic surgery in the management of submandibular gland sialoliths: a systematic review. J Clin Med. 2023;12:3007. 37109343 10.3390/jcm12083007 PMC10140901

[4] Zhao YN Zhang YQ Zhang LQ Xie XY Liu DG Yu GY. Treatment strategy of hilar and intraglandular stones in Wharton’s duct: a 12‐year experience. Laryngoscope. 2020;130:2360–5. 31691983 10.1002/lary.28361

[5] Koch M Schapher M Mantsopoulos K Goncalves M Iro H. Intraductal pneumatic lithotripsy after extended transoral duct surgery in submandibular sialolithiasis. Otolaryngol Head Neck Surg. 2019;160:63–9. 30296893 10.1177/0194599818802224

[6] Zenk J Constantinidis J Al-Kadah B Iro H. Transoral removal of submandibular stones. Arch Otolaryngol Head Neck Surg. 2001;127:432–6. 11296054 10.1001/archotol.127.4.432

[7] Capaccio P Gaffuri M Rossi V Pignataro L. Sialendoscope-assisted transoral removal of hilo-parenchymal sub-mandibular stones: surgical results and subjective scores= L’asportazione transorale scialoendoscopico-assistita dei calcoli ilo-parenchimali sottomandibolari: Risultati chirurgici e soggettivi. Acta Otorhinolaryngol Ital. 2017;37:122–7. 28516974 10.14639/0392-100X-1601 PMC5463519

[8] O’Brien CJ Murrant NJ. Surgical management of chronic parotitis. Head Neck. 1993;15:445–9. 8407318 10.1002/hed.2880150513

[9] Sadeghi N Black MJ Frenkiel S. Parotidectomy for the treatment of chronic recurrent parotitis. J Otolaryngol. 1996;25:305–7. 8902688

[10] Amin MA Bailey BMW Patel SR. Clinical and radiological evidence to support superficial parotidectomy as the treatment of choice for chronic parotid sialadenitis: a retrospective study. Br J Oral Maxillofac Surg. 2001;39:348–52. 11601814 10.1054/bjom.2001.0671

[11] Moody AB Avery CME Walsh S Sneddon K Langdon JD. Surgical management of chronic parotid disease. Br J Oral Maxillofac Surg. 2000;38:620–2. 11092780 10.1054/bjom.2000.0478

[12] Motamed M Laugharne D Bradley PJ. Management of chronic parotitis: a review. J Laryngol Otol. 2003;117:521–6. 12901804 10.1258/002221503322112923

[13] Koch M Zenk J Iro H. Algorithms for treatment of salivary gland obstructions. Otolaryngol Clin North Am. 2009;42:1173–92, Table of Contents. 19962014 10.1016/j.otc.2009.08.002

[14] Fabie JE Kompelli AR Naylor TM Nguyen SA Lentsch EJ Gillespie MB. Gland‐preserving surgery for salivary stones and the utility of sialendoscopes. Head Neck. 2019;41:1320–7. 30549387 10.1002/hed.25560

[15] Brandstätter W Hatzl M Weis S Javor A Gabriel M. Successful resection of TSH-secreting pituitary adenoma demonstrated by serial 99mTc-scintigraphy. Nuklearmedizin. 2015;54:N23–4. 26105720

[16] Chen C Guo P Chen X. Recurrent sublingual ranula or saliva leakage from the submandibular gland? Anatomical consideration of the ductal system of the sublingual gland. J Oral Maxillofac Surg. 2015;73:675. e1–675. e7. 10.1016/j.joms.2014.10.012 25795579

[17] Ashindoitiang JA Nwagbara VIC Ugbem T. Huge sialolith of the submandibular gland: a case report and review of literature. J Int Med Res. 2023;51:3000605221148443. 10.1177/03000605221148443 PMC9834782 36624984

[18] Mbalaso OC Nwogbo AC Uyanwanne NS. Huge submandibular gland calculus in Port Harcourt: a case report. Nigerian Health J. 2018;18:172–5.

[19] Koch M Mantsopoulos K Müller S Sievert M Iro H. Treatment of sialolithiasis: what has changed? An update of the treatment algorithms and a review of the literature. J Clin Med. 2021;11:231. 35011971 10.3390/jcm11010231 PMC8746135

[20] Luers JC Grosheva M Reifferscheid V Stenner M Beutner D. Sialendoscopy for sialolithiasis: early treatment, better outcome. Head Neck. 2012;34:499–504. 21484927 10.1002/hed.21762

[21] Iro H Zenk J Escudier MP. Outcome of minimally invasive management of salivary calculi in 4,691 patients. Laryngoscope. 2009;119:263–8. 19160432 10.1002/lary.20008

[22] Seward GR. Anatomic surgery for salivary calculi: part VII. Complications of salivary calculi. Oral Surg Oral Med Oral Pathol. 1968;26:1–7. 5242043 10.1016/0030-4220(68)90242-9

[23] Coumel C Vesse M Perrin L Rouaux JP. 50 submandibular gland resections (author’s transl). Rev Stomatol Chir Maxillofac. 1979;80:344–8. 294666

[24] Milton CM Thomas BM Bickerton RC. Morbidity study of submandibular gland excision. Ann R Coll Surg Engl. 1986;68:148–50. 3729264 PMC2498150

[25] Patton DW. Recurrent calculus formation following removal of the submandibular salivary gland. Br J Oral Maxillofac Surg. 1987;25:15–20. 2948540 10.1016/0266-4356(87)90152-5

[26] Berini-Aytes L Gay-Escoda C. Morbidity associated with removal of the submandibular gland. J Craniomaxillofac Surg. 1992;20:216–9. 1328305 10.1016/s1010-5182(05)80318-x

[27] Hald J Andreassen UK. Submandibular gland excision: short-and long-term complications. ORL J otorhinolaryngol Relat Spec. 1994;56:87–91. 8177591 10.1159/000276616

[28] Ellies M Laskawi R Arglebe C Schott A. Surgical management of nonneoplastic diseases of the submandibular gland: A follow-up study. Int J Oral Maxillofac Surg. 1996;25:285–9. 8910114 10.1016/s0901-5027(06)80058-5

[29] Kim EA Kim HS Kim HK. Recurrent sialolithiasis on remnant Wharton’s Duct following submandibular gland resection. Korean J Otorhinolaryngol Head Neck Surg. 2004;47:1185–7.

[30] Markiewicz MR Margarone JE 3rd Tapia JL Aguirre A. Sialolithiasis in a residual Wharton’s duct after excision of a submandibular salivary gland. J Laryngol Otol. 2007;121:182–5. 17076929 10.1017/S0022215106003525

[31] Ying X Kang J Zhang F Dong H. Recurrent sialoliths after excision of the bilateral submandibular glands for sialolithiasis treatment: A case report. Exp Ther Med. 2016;11:335–7. 26889264 10.3892/etm.2015.2849 PMC4726928

[32] Trujillo O Drusin MA Rahmati R. Rapid recurrent sialolithiasis: altered stone composition and potential factors for recurrence. Laryngoscope. 2017;127:1365–8. 27753112 10.1002/lary.26357

[33] Harrison JD. Causes, natural history, and incidence of salivary stones and obstructions. Otolaryngol Clin North Am. 2009;42:927–47. 19962002 10.1016/j.otc.2009.08.012

[34] Tanaka N Ichinose S Adachi Y Mimura M Kimijima Y. Ultrastructural analysis of salivary calculus in combination with X-ray microanalysis. Med Electron Microsc. 2003;36:120–6. 12825126 10.1007/s00795-002-0210-z

[35] Mimura M Tanaka N Ichinose S Kimijima Y Amagasa T. Possible etiology of calculi formation in salivary glands: biophysical analysis of calculus. Med Mol Morphol. 2005;38:189–95. 16170467 10.1007/s00795-005-0290-7

[36] Zheng LY Kim E Yu CQ Yang C Park J Chen ZZ. A retrospective case series illustrating a possible association between a widened hilum and sialolith formation in the submandibular gland. J Craniomaxillofac Surg. 2013;41:648–51. 23375532 10.1016/j.jcms.2013.01.001

[37] Makdissi J Escudier MP Brown JE Osailan S Drage N McGurk M. Glandular function after intraoral removal of salivary calculi from the hilum of the submandibular gland. Br J Oral Maxillofac Surg. 2004;42:538–41. 15544884 10.1016/j.bjoms.2004.08.006

[38] Austin T Davis J Chan T. Sialolithiasis of submandibular gland. J Emerg Med. 2004;26:221–3. 14980352 10.1016/j.jemermed.2003.07.007

[39] Grases F Santiago C Simonet BM Costa-Bauzá A. Sialolithiasis: mechanism of calculi formation and etiologic factors. Clin Chim Acta. 2003;334:131–6. 12867283 10.1016/s0009-8981(03)00227-4

[40] Su Y Zhang K Ke Z Zheng G-sen Chu M Liao G-qing. Increased calcium and decreased magnesium and citrate concentrations of submandibular/sublingual saliva in sialolithiasis. Arch Oral Biol. 2010;55:15–20. 19962126 10.1016/j.archoralbio.2009.11.006

[41] Epivatianos A Harrison JD Dimitriou T. Ultrastructural and histochemical observations on microcalculi in chronic submandibular sialadenitis. J Oral Pathol. 1987;16:514–7. 3127566 10.1111/j.1600-0714.1987.tb00683.x

[42] Epivatianos A Harrison JD. The presence of microcalculi in normal human submandibular and parotid salivary glands. Arch Oral Biol. 1989;34:261–5. 2597019 10.1016/0003-9969(89)90066-6

[43] Scott J. The prevalence of consolidated salivary deposits in the small ducts of human submandibular glands. J Oral Pathol. 1978;7:28–37. 418161 10.1111/j.1600-0714.1978.tb01882.x

[44] Harrison JD Epivatianos A Bhatia SN. Role of microliths in the aetiology of chronic submandibular sialadenitis: a clinicopathological investigation of 154 cases. Histopathology. 1997;31:237–51. 9354894 10.1046/j.1365-2559.1997.2530856.x

